# Prevalence of syphilis and chlamydia trachomatis infection among men who have sex with men in Jiangsu province, China: A cross-sectional survey

**DOI:** 10.3389/fpubh.2022.1006254

**Published:** 2022-10-11

**Authors:** Haiyang Hu, Yuheng Chen, Lingen Shi, Xiaoxia Liu, Zhuping Xu, Lin Sun, Xiuping Zhao, Ying Zhou, Jing Lu, Zhi Zhang, Xiaoyan Liu, Gengfeng Fu

**Affiliations:** ^1^Jiangsu Provincial Center for Disease Control and Prevention, Nanjing, China; ^2^Zhenjiang Center for Disease Control and Prevention, Zhenjiang, China; ^3^Wuxi Center for Disease Control and Prevention, Wuxi, China; ^4^Yangzhou Center for Disease Control and Prevention, Yangzhou, China; ^5^Suzhou Center for Disease Control and Prevention, Suzhou, China

**Keywords:** syphilis, chlamydia, prevalence, men who have sex with men (MSM), China

## Abstract

**Background:**

Epidemics of sexually transmitted infections (STIs) among men who have sex with men (MSM) are major global public health concerns. This study aimed to examine the prevalence of syphilis and chlamydia trachomatis (CT) infection and associated factors among MSM in Jiangsu province, China, hoping to provide updated data for the formulation of relevant policies.

**Methods:**

A cross-sectional survey was conducted among MSM from April to July 2021 in four cities in the province. Socio-demographic characteristics and behavioral information were collected through a face-to-face questionnaire interview. Venous blood specimens were collected for HIV, hepatitis C (HCV), and syphilis testing using serological testing methods. First-void urine specimens were collected for CT and Neisseria gonorrhoeae (NG) testing using nucleic acid amplification testing (NAAT) methods. Chi-square tests were used to compare differences in syphilis and CT infection between subgroups of variables. Multivariate logistic regression analysis was used to identify factors associated with syphilis and CT infection.

**Results:**

A total of 1,087 participants were enrolled. The prevalence of HIV, HCV, syphilis, CT and NG infection were 6.6, 0.4, 6.3, 4.2, and 0.4%, respectively. MSM recruited online [adjusted odds ratio (aOR) = 2.189, *P* = 0.020], diagnosed with an STI in the past 12 months (aOR = 3.304, *P* < 0.001), and living with HIV (aOR = 4.721, *P* < 0.001) were more likely to have syphilis infection. MSM who were younger than 25 years (aOR = 4.286, *P* = 0.020), had senior high school level education (aOR = 2.521, *P* = 0.038), and were recruited *via* VCT clinics (aOR = 3.455, *P* = 0.001) were more likely to have CT infection.

**Conclusions:**

Our study showed a high prevalence of syphilis and chlamydia among MSM in Jiangsu province, China. STI screening, diagnosis, and treatment services promotion should be a top priority on the prevention agenda.

## Introduction

Sexually transmitted infections (STIs) are among the most common infectious diseases globally and affect the health and lives of people worldwide. According to a report by the World Health Organization (WHO), there were about 373.1 million STI infections (four of the most common curable STIs) in 2020, of which 128 million chlamydia cases, 82 million gonorrhea cases, 156 million trichomoniasis cases, and 7.1 million syphilis cases (1).

These STIs can cause acute urogenital diseases like urethritis, vaginitis, cervicitis, and genital ulceration. Also, their etiological agents sometimes infect the rectum and pharynx. Chlamydia (etiological agent: Chlamydia trachomatis, CT) and gonorrhea (Neisseria gonorrhoeae, NG) can cause serious complications, including pelvic inflammatory disease in women and orchitis in men. Syphilis (etiological agent: Treponema pallidum) can cause dermatological, neurological, and cardiovascular diseases (2, 3). These STIs are curable but can increase the risk of human immunodeficiency virus (HIV) infection, especially in men who have sex with men (MSM), the group most at risk of HIV infection (4–6).

CT, NG, and syphilis infections were associated with an increased risk of HIV infection in men, possibly due to mucosal inflammation and ulceration of local tissues leading to HIV invasion (7). MSM with HIV and urethral STIs may also have higher viral loads of HIV in semen and thus be more likely to transmit HIV to their sexual partners during unprotected anal intercourse (8). Although direct studies have not established a relationship between STI treatment and reduced HIV transmission rates, urethritis treatment in HIV infection may reduce seminal plasma viral load (9). Model studies and at least one randomized study showed that treatment of STIs may reduce HIV transmission (10, 11). However, CT and syphilis infections are sometimes asymptomatic, and infected individuals may not be screened during routine clinical evaluation. This leads to late diagnosis leading to persistent, unintentional STI and HIV transmission (12).

Understanding the prevalence of STIs among MSM for HIV prevention and control is important. A nationwide cross-sectional study conducted in 61 cities in China from February 2008 to September 2009 found that 11.8% of MSM had syphilis infection (13). In a survey involving MSM in two cities in Jiangsu province of China in 2009, the prevalence of STIs was measured, including CT (6.54%), NG (3.63%), and syphilis (20.34%) (6). In 2010, the Chinese government launched a plan to prevent and control syphilis. Many initiatives focused on prevention and control measures that expanded syphilis testing uptake among key populations, including MSM, and early detection and treatment of syphilis infections. A spatiotemporal meta-analysis of syphilis epidemic among men who have sex with men living in mainland China showed that national syphilis prevalence decreased from 12.3% in 2001–2007 to 7.1% in 2013–2015 (14). However, by the end of 2020, reports on CT prevalence and measures on prevention among MSM were few. Located in southeastern China, Jiangsu province is economically developed, with a more than 80 million population. Herein, we conducted this cross-sectional survey to examine the prevalence of syphilis and CT infection and their associated factors among MSM in Jiangsu province, China, to provide more recent data for formulating relevant policies.

## Materials and methods

### Study design and participants

A cross-sectional survey was conducted among MSM at the AIDS and STD surveillance sites in Jiangsu province from April to July 2021. MSM were enrolled in one of four cities (Zhenjiang, Wuxi, Yangzhou, and Suzhou) in the province that acted as survey sites for the study. Eligible participants were assigned male gender at birth, 18 years of age or older, and self-reported anal or oral sex with another male in the previous year. Each recruited participant had a face-to-face questionnaire interview and specimen collection. All participants provided written informed consent before enrolment. Implementation of AIDS and STD surveillance sites is a routine part of disease control and prevention, so this study was exempt from ethical review.

### Measures

Three convenience sampling methods were used to recruit participants: (1) Recruitment at MSM gathering venues: Staff from local centers for disease control and prevention (CDCs) and volunteers from local MSM-led community-based organizations (CBOs) conducted on-site surveys at popular MSM gathering venues. Venue owners (e.g., bars, clubs, and bathhouses) and volunteers who knew of public places where MSM frequently gathered (e.g., parks or public restrooms) referred interested and eligible participants to the interviewers.

(2) Online recruitment: Volunteers posted recruitment information, including eligibility criteria, survey sites, survey period, and contact numbers through WeChat, QQ, and other apps. Eligible participants came to the designated location to complete the questionnaire and specimen collection.

(3) Recruitment at VCT clinics: Eligible MSM clients attending VCT (HIV voluntary counseling and testing) clinics were recruited for enrollment in the study after receiving counseling from the VCT clinic staff.

The interviews remained anonymous throughout the study, and any information that could identify individual participants was exempted from the final data. Participants' cell phone numbers were obtained for testing results notification and referrals to infection-related resources and services. In order to prevent duplicate participation, same interviewers were staffed at each study site to identify participants (e.g., face, special characteristics). In addition, staff asked participants if they already participated in similar surveys, and used computers to check participants' cell phone numbers for duplication before the interviews. In our study, there were no duplicate participation between venue, online and VCT recruitment.

Participants were interviewed face-to-face by interviewers who completed provincial or municipal surveillance training, especially in STI training. The information obtained with the questionnaire included socio-demographic characteristics (age, marital status, education level, registered residence, duration of living locally, and monthly income) and STI-related behavior (sexual orientation, the main way to find a sexual partner, unprotected anal intercourse in the past 6 months, STI in the past 12 months and HIV testing in the past 12 months). After completing questionnaire interviews by interviewers, the quality controllers who completed training courses checked the quality of the questionnaire. Unprotected anal intercourse (UAI) was defined as inconsistent condom use with male partners. Not having anal sex was equivalent to consistent condom use.

After questionnaire interviews, venous blood specimens collected from each participant were tested for HIV, Hepatitis C virus (HCV), and syphilis using serological testing methods. Plasma HIV and HCV antibodies were tested using an enzyme-linked immunosorbent assay (ELISA) reagent (Zhuhai livzon Diagnostics Inc., Zhuhai, China). For those with positive HIV or HCV test results, the same blood samples were retested with another ELISA reagent (Beijing Wantai Biological Pharmacy Enterprise Co., Ltd., Beijing, China). If both tests were positive, participants were contacted by CDC staff through reserved phone numbers. Participants with positive HCV results were referred to infectious disease hospitals for treatment and care. Participants with positive HIV results by ELISA testing provided new blood specimens for HIV confirmatory testing by western blot assay (MP Biomedical Asia Pacific Pte. Ltd., Singapore). The previous blood samples were used for confirmatory testing if participants were lost to follow-up after the initial testing. The syphilis antibody testing was conducted using an ELISA reagent (Beijing Wantai) and confirmed using a toluidine red untreated serum test (TRUST, Beijing Wantai). Syphilis infection was defined as having two positive tests, and participants were referred to designated facilities for treatment. First-void urine specimens were collected from each participant to test for CT and NG with nucleic acid amplification testing (NAAT) methods (Shanghai Rendu Biotechnology Co., Ltd., Shanghai, China). Auto-SAT automatic nucleic acid testing and analysis system (Shanghai Rendu) was used to test urine specimens for CT and NG nucleic acid. If tests were positive, participants were considered as having CT or NG infection.

### Statistical analysis

Questionnaire data at each survey site were double-entered and checked for accuracy using EpiData software (version 3.1). Quantitative variables were grouped, and qualitative variables were merged according to the data distribution. Socio-demographic and STI-related behavioral characteristics of participants were descriptively analyzed using frequencies. Chi-square tests were used to compare syphilis and CT infection differences between subgroups of variables to seek potential associated factors. Variables with *P*-values < 0.20 were entered into multivariable logistic regression models to identify independent factors associated with syphilis and CT infection using a forward method to determine the adjusted odds ratios (aORs). *P*-values < 0.05 were considered statistically significant. All analyses were conducted using SPSS software (version 19.0).

## Results

### Socio-demographic characteristics of participants

A total of 1,087 eligible participants were enrolled in the study per our informed consent, completed questionnaire, and laboratory testing protocol. The average age of participants was 29.6, with a standard deviation of 12.2 (range: 18–77). Participants aged 24 years or younger constituted 45.0% (489), and those aged 25–39 constituted 37.2% (404). About three-quarters of participants (74.8%, 813) were single, divorced, or widowed. Participants' distribution across the three levels of education was similar (30.8, 35.0, and 34.2%, respectively). Most participants (57.9%, 629) were registered residents of Jiangsu province and had lived locally for more than 2 years (56.3%, 612). Participants with a monthly income of <5,000 CNY (China Yuan) and more than 5,000 CNY were equally represented. 64.9% (706) of participants were recruited from MSM gathering venues, and others were recruited online (17.4%, 189) and at VCT clinics (17.7%, 192) (Table 1).

**Table 1:** Socio-demographic characteristics of 1,087 MSM in a cross-sectional survey in Jiangsu province, China.

**Variables**	**No. (%)**
**Age group (years)**
<25	489 (45.0)
25–39	404 (37.2)
≥40	194 (17.8)
**Marital status**
Single, divorced or widowed	813 (74.8)
Married or cohabiting	274 (25.2)
**Education level**
Junior high school or below	335 (30.8)
Senior high school	380 (35.0)
College or above	372 (34.2)
**Registered residence**
Jiangsu province	629 (57.9)
Other provinces	458 (42.1)
**Duration of living locally**
<2 years	475 (43.7)
≥2 years	612 (56.3)
**Monthly income (CNY)**
<5,000	549 (50.5)
≥5,000	538 (49.5)
**Recruitment source**
Venues	706 (64.9)
Internet	189 (17.4)
VCT clinic	192 (17.7)

MSM, men who have sex with men; VCT, HIV voluntary counseling and testing; CNY, China Yuan.

### STI-related behavioral characteristics of participants

Among participants, 41.9% (455) identified as homosexuals, 25.6% (278) were heterosexuals, and 32.6% (354) were bisexuals (self-reported). 38.5% (418) of participants found sexual partners at MSM gathering venues, and 61.5% (669) did through the Internet or dating apps. Only 12.8% (139) engaged in UAI in the past 6 months, 7.3% (79) were diagosed with an STI within the past 12 months, and the majority (55.7%, 605) tested for HIV in the last 12 months (Table 2).

**Table 2:** STI-related behaviors of participants.

**Variables**	**No. (%)**
**Sexual orientation**
Homosexual	455 (41.9)
Heterosexual	278 (25.6)
Bisexual or indetermination	354 (32.6)
**The main way to find a sexual partner**
MSM venue	418 (38.5)
Internet or dating app	669 (61.5)
**UAI in the past 6 months**
No	948 (87.2)
Yes	139 (12.8)
**STI in the past 12 months**
No	1,008 (92.7)
Yes	79 (7.3)
**HIV testing in the past 12 months**
No	482 (44.3)
Yes	605 (55.7)

STI, Sexually transmitted infection; UAI, Unprotected anal intercourse.

### Prevalence of syphilis, CT, and other infectious diseases

Among the 1,087 participants, 69 tested positive for syphilis, showing a high prevalence of 6.3% [95% confidence interval (CI): 4.9–7.8%]. Forty six tested positive for CT infection with a prevalence of 4.2% (95% CI: 3.0–5.4%). HIV, HCV, and NG infection prevalence were 6.6% (95% CI: 5.1–8.1%, *n* = 72), 0.4% (0–0.7%, *n* = 4), and 0.4% (0–0.7%, *n* = 4), respectively. Besides, 1.5% (*n* = 16) had HIV-syphilis coinfection; two had syphilis-CT coinfection; one had HIV-HCV coinfection; no HIV-CT coinfection was found.

### Factors associated with syphilis and CT infection

Chi-square tests were used to compare syphilis and CT infection differences between subgroups of variables. The results showed that syphilis was significantly associated with UAI in the past 6 months, STI diagnosis in the past 12 months, and HIV infection (all *P* < 0.05). CT infection was significantly associated with age, education level, registered residence, duration of living locally, recruitment source, sexual orientation, UAI in the past 6 months, and HIV testing in the past 12 months (all *P* < 0.05) (Table 3).

**Table 3:** Differences in syphilis and CT infection between subgroups of variables among men who have sex with men.

**Variables**	**Syphilis infection**			**CT infection**		
	**No. positive (%)**	**No. negative (%)**	***P*-value**	**No. positive (%)**	**No. negative (%)**	***P*-value**
Age group (years)			0.418			0.000
<25	27 (39.1)	462 (45.4)		35 (76.1)	454 (43.6)	
25–39	26 (37.7)	378 (37.1)		8 (17.4)	396 (38.0)	
≥40	16 (23.2)	178 (17.5)		3 (6.5)	191 (18.3)	
Marital status			0.862			0.052
Single, divorced or widowed	51 (73.9)	762 (74.9)		40 (87.0)	773 (74.3)	
Married or cohabiting	18 (26.1)	256 (25.1)		6 (13.0)	268 (25.7)	
Education level			0.914			0.039
Junior high school or below	22 (31.9)	313 (30.7)		16 (34.8)	319 (30.6)	
Senior high school	25 (36.2)	355 (34.9)		22 (47.8)	358 (34.4)	
College or above	22 (31.9)	350 (34.4)		8 (17.4)	364 (35.0)	
Registered residence			0.815			0.003
Jiangsu province	39 (56.5)	590 (58.0)		17 (37.0)	612 (58.8)	
Other provinces	30 (43.5)	428 (42.0)		29 (63.0)	429 (41.2)	
Duration of living locally			0.970			0.003
<2 years	30 (43.5)	445 (43.7)		30 (65.2)	445 (42.7)	
≥2 years	39 (56.5)	573 (56.3)		16 (34.8)	596 (57.3)	
Monthly income (CNY)			0.146			0.330
<5,000	29 (42.0)	520 (51.1)		20 (43.5)	529 (50.8)	
≥5,000	40 (58.0)	498 (48.9)		26 (56.5)	512 (49.2)	
Recruitment source			0.112			0.043
Venues	37 (53.6)	669 (65.7)		30 (65.2)	676 (64.9)	
Internet	17 (24.6)	172 (16.9)		3 (6.5)	186 (17.9)	
VCT clinic	15 (21.7)	177 (17.4)		13 (28.3)	179 (17.2)	
Sexual orientation			0.582			0.000
Homosexual	31 (44.9)	424 (41.7)		11 (23.9)	444 (42.7)	
Heterosexual	14 (20.3)	264 (25.9)		23 (50.0)	255 (24.5)	
Bisexual or indetermination	24 (34.8)	330 (32.4)		12 (26.1)	342 (32.9)	
The main way to find a sexual partner			0.891			0.051
MSM venue	26 (37.7)	392 (38.5)		24 (52.2)	394 (37.8)	
Internet or dating app	43 (62.3)	626 (61.5)		22 (47.8)	647 (62.2)	
UAI in the past 6 months			0.002			0.028
No	52 (75.4)	896 (88.0)		45 (97.8)	903 (86.7)	
Yes	17 (24.6)	122 (12.0)		1 (2.2)	138 (13.3)	
STI in the past 12 months			0.000			0.375*
No	55 (79.7)	953 (93.6)		41 (89.1)	967 (92.9)	
Yes	14 (20.3)	65 (6.4)		5 (10.9)	74 (7.1)	
HIV testing in the past 12 months			0.919			0.009
No	31 (44.9)	451 (44.3)		29 (63.0)	453 (43.5)	
Yes	38 (55.1)	567 (55.7)		17 (37.0)	588 (56.5)	
HIV infection			0.000*			0.068*
No	53 (76.8)	962 (94.5)		46 (100.0)	969 (93.1)	
Yes	16 (23.2)	56 (5.5)		0 (0.0)	72 (6.9)	
Syphilis infection			–			0.763*
No	–	–		44 (95.7)	974 (93.6)	
Yes	–	–		2 (4.3)	67 (6.4)	
CT infection			0.763*			–
No	67 (97.1)	974 (95.7)		–	–	
Yes	2 (2.9)	44 (4.3)		–	–	

^*^Fisher's precision probability test. CT, Chlamydia trachomatis; VCT, HIV voluntary counseling and testing; UAI, Unprotected anal intercourse; STI, Sexually transmitted infection; CNY, China Yuan.

In the multivariate logistic regression analysis, MSM who were recruited online (aOR = 2.189, 95% CI: 1.133–4.230, *P* = 0.020), diagnosed with an STI in the past 12 months (aOR = 3.304, 95% CI: 1.696–6.436, *P* < 0.001), and were infected with HIV (aOR = 4.721, 95% CI: 2.404–9.271, *P* < 0.001) were more likely to have syphilis infection. MSM who were younger than 25 years (aOR = 4.286, 95% CI: 1.259–14.587, *P* = 0.020), had a senior high school level of education (aOR = 2.521, 95% CI: 1.050–6.052, *P* = 0.038), and were recruited at VCT clinics (aOR = 3.455, 95% CI: 1.658–7.200, *P* = 0.001) were more likely to have CT infection (Table 4).

**Table 4:** Factors independently associated with syphilis and CT infection among men who have sex with men in Jiangsu province, China.

**Variables**	**Syphilis infection**		**CT infection**	
	**aOR (95% CI)**	***P*-value**	**aOR (95% CI)**	***P*-value**
**Age group (years)**
<25			4.286 (1.259–14.587)	0.020
25–39			1.419 (0.361–5.587)	0.617
≥40			1.000	
**Education level**
Junior high school or below			1.818 (0.713–4.640)	0.211
Senior high school			2.521 (1.050–6.052)	0.038
College or above			1.000	
**Recruitment source**
Venues	1.000		1.000	
Internet	2.189 (1.133–4.230)	0.020	0.622 (0.178–2.172)	0.457
VCT clinic	1.033 (0.523–2.039)	0.925	3.455 (1.658–7.200)	0.001
**STI in the past 12 months**
No	1.000			
Yes	3.304 (1.696–6.436)	0.000		
**HIV infection**
No	1.000			
Yes	4.721 (2.404–9.271)	0.000		

CT, Chlamydia trachomatis; VCT, HIV voluntary counseling and testing; STI, Sexually transmitted infection.

## Discussion

In recent years, MSM have become the group at highest risk of STI infection worldwide, higher than female sex workers and much higher than the general population. According to a survey conducted in 61 cities in China from 2008 to 2009, the prevalence of syphilis among MSM was 11.8% (13). Data from the National AIDS sentinel surveillance program showed that the prevalence of syphilis among MSM decreased from 8.6% in 2010 to 6.4% in 2013 (15). A consecutive survey conducted in Jiangsu province showed that the prevalence of syphilis among MSM was 10.2% in 2011 (16), 8.3% in 2015 (17), and 6.3% in our study, showing a significant decline. Our observed declining trend is similar to that of the world (18), China (15), and parts of China (19, 20). A meta-analysis of syphilis incidence among MSM in China yielded similar results (21). As a curable STI, the expansion of syphilis screening, diagnosis, and treatment services play an important role in preventing and controlling the disease (18). Studies on syphilis self-testing among MSM have been conducted in parts of China to evaluate the expansion of accessibility to syphilis prevention services (22, 23).

In our study, the prevalence of urogenital CT infection among MSM was 4.2%. This rate was similar to the prevalence found in Thailand (24), lower than that in Guangzhou, China (25), far lower than that in Papua New Guinea (26), and higher than that in Germany (27) and Wuhan of China (28). In addition, there has been no obvious decreasing trend in the prevalence of CT infection, especially in China. Compared to the syphilis prevention and control strategy, there is no expanded CT screening strategy for high-risk groups in China, which needs to be strengthened in the future. Our study showed a low prevalence of urogenital NG infection among MSM, far lower than that in two other China-based studies (25, 28). Differences in the MSM recruitment sources may explain the contradictions in our findings. In the other two studies, MSM participants were recruited from STI clinics (25, 28), whereas MSM were recruited from MSM gathering venues, online, and VCT clinics in our study. Unlike CT infection, which is mostly asymptomatic, urogenital NG infection in men often presents acute urethral inflammation, which could prompt early testing and treatment. This may be another possible reason for the huge differences in NG infection rates.

Consistent with the findings of some studies (17, 29), HIV infection and online recruitment were independent risk factors for syphilis among MSM in our study. MSM recruited online may use online spaces to find sexual partners more often, increasing their risk of syphilis infection due to multiple sexual partners and reduced self-protection awareness. Therefore, we should pay more attention to this group of MSM, promoting more frequent testing and intervention of syphilis. HIV infection is a risk factor for syphilis infection, which shows that these two diseases are closely related. Interventions for encouraging condom use should be strengthened for people living with HIV. They are less likely to transmit HIV to others after effective antiretroviral treatment but more likely to transmit STIs like syphilis. The prevention and control of STIs and AIDS should be integrated and implemented as a policy.

We found that age, education level, and recruitment source were factors independently associated with CT infection. In line with some studies (25, 28, 29), young MSM are more likely to have CT infection due to more engagement in UAI and limited STI prevention knowledge or awareness. Therefore, there is a need to pay more attention to STI-related health education among MSM, and special efforts should be made to provide services to young MSM. Compared to MSM with college or above education, MSM with senior high school level of education are more likely to be CT infected. There are differences in knowledge of STI prevention and awareness of the need for regular testing between MSM with different levels of education. The health education and testing service for this particular group should be strengthened. MSM in VCT clinics usually have high sexual-risk behaviors, so this group has a high risk of CT infection. Although UAI was not independently associated with syphilis and CT infection in our study, it is well known that UAI is a risk factor for STIs in MSM. Therefore, promoting condom use would still play an important role in preventing and controlling these STIs' transmission.

Through this study, we have determined the current prevalence of syphilis and CT infection among MSM in Jiangsu province and possible risk factors, which are very important for future STI prevention and control. However, several limitations of this study should be noted. First, we did not rely on the STI clinic to recruit MSM. So, we only collected convenient urine specimens and did not collect rectal specimens, which may have shown a higher CT positive rate. Nonetheless, we will continue to observe trends in CT infection rates through multi-year surveys. Second, due to memory bias and face-to-face interview survey patterns, participants might have provided incorrect, fragmentary, or socially expected answers. Third, our study was conducted among some MSM in Jiangsu province, and our results may not be generalizable to all MSM in the province and other regions. Besides, this study was done during the COVID-19 pandemic period. The composition of participants may be affected, for example by the enclosed management of some universities.

## Conclusions

This study showed a high prevalence of syphilis and chlamydia among MSM in Jiangsu province, China. Therefore, it is necessary to strengthen the screening and treatment of STIs in MSM to improve their sexual health.

## Data availability statement

The raw data supporting the conclusions of this article will be made available by the authors, without undue reservation.

## Ethics statement

The studies involving human participants were reviewed and approved by Institutional Review Board of Jiangsu Provincial Center for Disease Control and Prevention. The patients/participants provided their written informed consent to participate in this study.

## Author contributions

HH, YC, and GF contributed to conception and design of the study. YC, LSh, XiaoxL, ZX, LSu, and XZ organized the database. HH and YC performed the statistical analysis. HH wrote the first draft of the manuscript. YZ, JL, ZZ, and XiaoyL wrote sections of the manuscript. All authors contributed to manuscript revision, read, and approved the submitted version.

## Conflict of interest

The authors declare that the research was conducted in the absence of any commercial or financial relationships that could be construed as a potential conflict of interest.

## Publisher's note

All claims expressed in this article are solely those of the authors and do not necessarily represent those of their affiliated organizations, or those of the publisher, the editors and the reviewers. Any product that may be evaluated in this article, or claim that may be made by its manufacturer, is not guaranteed or endorsed by the publisher.
